# Multidrug-Resistant and Extensively Drug-Resistant Tuberculosis in Multi-Ethnic Region, Xinjiang Uygur Autonomous Region, China

**DOI:** 10.1371/journal.pone.0032103

**Published:** 2012-02-22

**Authors:** Ying-Cheng Qi, Mai-Juan Ma, Dong-Jun Li, Mei-Juan Chen, Qing-Bin Lu, Xiu-Jun Li, Jun-Lian Li, Wei Liu, Wu-Chun Cao

**Affiliations:** 1 Department of Tuberculosis, Chest Hospital of Xinjiang Uygur Autonomous Region, Urumqi, People's Republic of China; 2 Department of Epidemiology, Beijing Institute of Microbiology and Epidemiology, State Key Laboratory of Pathogen and Biosecurity, Beijing, People's Republic of China; 3 Emergency Center of Red Cross Aid Center of Urumqi, Urumqi, People's Republic of China; 4 Department of Epidemiology and Health Statistics, Shandong University, Jinan, People's Republic of China; St. Petersburg Pasteur Institute, Russian Federation

## Abstract

**Background:**

The multidrug-resistant (MDR) and extensively drug-resistant (XDR) tuberculosis (TB) has emerged as a global threat. Xinjiang is a multi-ethnic region and suffered second highest incidence of TB in China. However, epidemiological information on MDR and XDR TB is scarcely investigated.

**Methodology/Principal Findings:**

A prospective study was conducted to analyze the prevalence of MDR and XDR TB and the differences of drug resistance TB between Chinese Han and other nationalities population at Chest Hospital of Xinjiang Uygur Autonomous Region, China. We performed in vitro drug susceptibility testing of *Mycobacterium tuberculosis* to first- and second-line anti-tuberculosis drugs for all 1893 culture confirmed positive TB cases that were diagnosed between June 2009 and June 2011. Totally 1117 (59.0%, 95% CI, 56.8%–61.2%) clinical isolates were resistant to ≥1 first-line drugs; the prevalence of MDR TB was 13.2% (95% CI, 11.7%–14.7%), of which, 77 (30.8%; 95% CI, 25.0%–36.6%) and 31 (12.8%; 95% CI, 8.6%–17.0%) isolates were pre-XDR and XDR TB respectively. Among the MDR/XDR TB, Chinese Han patients were significantly less likely to be younger with an odds ratio 0.42 for age 20–29 years and 0.52 for age 40–49 years; *P*_trend_ = 0.004), and Chinese Han patients has a lower prevalence of XDR TB (9.6%) than all the other nationality (14.9%).

**Conclusions/Significance:**

The burden of drug resistance TB cases is sizeable, which highlights an urgent need to reinforce the control, detection and treatment strategies for drug resistance TB. However, the difference of MDR and XDR TB between Chinese Han and other nationalities was not observed.

## Introduction

A third of the world's population is estimated to be infected with *Mycobacterium tuberculosis* (*M.tuberculosis*) according to the report of World Health Organization [1]. The emergence and spread of multidrug-resistant (MDR) and extensively drug-resistant (XDR) tuberculosis (TB) is further hampering efforts to control and manage the disease. Since the emergence of MDR strains in the 1990s, the prevalence of MDR TB has constantly increased. In 2008, about 440 000 MDR TB cases occurred in the world, leading to an estimated 150 000 deaths. XDR TB is a nearly untreatable form of the disease, and has been reported in 58 countries [2] with an estimated rate of 15% among the MDR strains [3].

TB is a significant public health problem in mainland China with an incidence around 100 per 100 000 populations [1]. Owning to the implementation of DOT'S plan, TB prevalence used to be decreased by 30% [4]. However, the emergence of drug resistance has severely threatened TB control in the country, and has raised the concern of a return to an era in which drugs are no longer effective [5]. According to National Baseline Survey on Drug-resistant Tuberculosis in mainland China during 2007–2008, 8.3% pulmonary TB patients were MDR based on the drug susceptibility tests of *M. tuberculosis* isolates [6].

Xinjiang Uygur Autonomous Region has the second highest incidence of TB among all the provinces and Autonomous Regions in China. The estimated prevalence of active pulmonary TB is 464 per 100 000, which might be caused by the spread of the HIV infection, increasing of floating population, poor health practices and bad living conditions in rural area, and with approximate 6700 deaths each year [7]. Xinjiang is also a multi-ethnic region, with minorities other than Han population accounting for 61.01% of the total population by the end of 2009. The difference of TB incidence used to be observed between ethnics [8], however, information on the prevalence of MDR and XDR TB remain scant in the region, since the first and second-line anti-TB drug susceptibility testing (DST) was not performed as a routine test in most of the local hospitals and the available data were not collected in a proper way to reflect the situation of the MDR/XDR TB occurrence in the region. This has hindered the informed efforts of disease control. The objectives of the study were to estimate the prevalence of MDR and XDR TB, and to investigate the differences in the drug-resistant profiles of TB among various ethnic groups.

## Results

### Demographics of the study subjects

**Figure 1:**
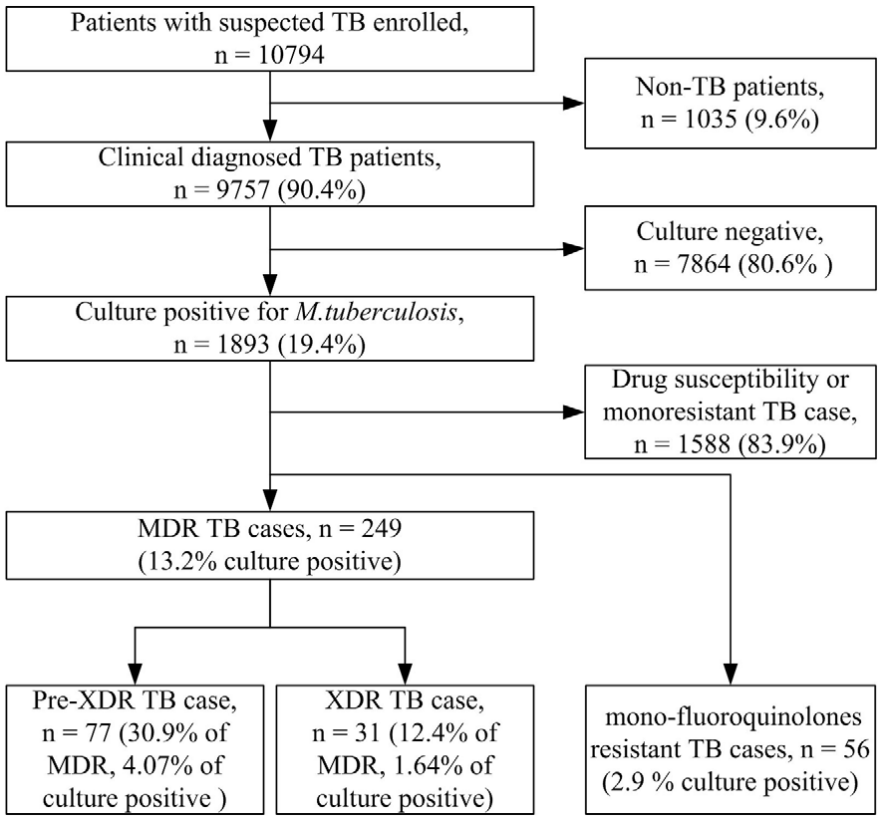
Determination of prevalence of tuberculosis (TB) and drug resistance among persons with suspected TB, Xinjiang Uygur Autonomous Region, China, during June 2009–June 2011. MDR, multidrug-resistant; XDR, extensively drug-resistant.

During June 2009 to June 2011, a total of 9759 clinically diagnosed TB patients were enrolled at Chest Hospital of Xinjiang Uygur Autonomous Region (CHXUAR), providing their sputa for *M. tuberculosis* test. Of these 9759 TB patients, 2518 (25.8%) had a positive acid-fast bacilli smear, and 7864 (80.6%) had a negative culture. As a result, 1893 (19.4%) patients with culture positive sputum were enrolled for anti-TB DST (figure 1). The age of the 1893 patients ranged from 2 to 95 years (mean (±SD), 43.0±19.1, media 40),57.2% of them were male and 48.5% of them were Chinese Han people, and the others belonged to other nationality groups. Majorities (85.5%) of them were inpatients and 37.4% were newly diagnosed cases. All of the patients were negative for HIV infection.

### Resistance to the first-line drugs

Out of the 1893 *M. tuberculosis* isolates, 1117 (59.0%; 95% CI, 56.8%–61.2%) were resistant to at least 1 first-line drug, 36.2% (95% CI, 34.0%–38.4%) were resistant to streptomycin, 36.1% (95% CI, 33.9%–38.2%) to isoniazid, 19.4% (95% CI, 17.7%–21.3%) to rifampin, and 17.5% (95% CI, 15.8%–19.3%) to ethambutol (table 1). Isolates from 249 patients were resistant to both isoniazid and rifampin, resulting in a MDR TB rate of 13.2% (95% CI, 11.6%–14.7%), which contain 89 newly diagnosed patients with MDR rate of 12.6% and 160 re-treatment patients with MDR rate of 13.5%. Of the 249 MDR isolates, 176 (70.7%) demonstrated resistance to other first-line drugs, including 111 (44.6%) were resistant to streptomycin, 23 (9.2%) resistant to ethambutol and 43 resistant (17.2%) to both.

**Table 1:** First and second line drug resistant frequency among 1893 clinical *Mycobacterium tuberculosis* isolates, June 2009–June 2011, Xinjiang Uygur Autonomous Region, China.

Drugs	No. isolates	Rate, % (95% CI)
Overall first-line drug resistance	1117	59.0(56.8–61.2)
INH	683	36.1(33.9–38.2)
RFP	368	19.4(17.7–21.2)
SM	685	36.2(34.0–38.4)
EMB	332	17.5(15.8–19.3)
Overall MDR	249	13.2(11.6–14.7)
INH+RFP	73	3.9(3.0–4.7)
INH+RFP+SM	111	5.9(4.8–6.9)
INH+RFP+EMB	23	1.2(0.7–1.7)
INH+RFP+SM+EMB	42	2.2(1.6–2.9)
Overall second-line drug resistance	616	32.5(30.4–34.7)
FQ	477	25.2(23.2–27.2)
AMK	113	6.0(5.0–7.0)
CPM	121	6.4(5.3–7.5)
KAN	169	8.9(7.6–10.2)
PTA	85	4.5(3.6–5.4)
Overall pre-XDR	77	30.9(25.1–36.7)
FQ	65	26.1(20.6–31.6)
AMK	6	2.4(0.5–4.3)
CPM	2	0.8*
KAN	4	1.6*
Overall XDR	31	12.5(8.3–16.6)
FQ+AMK	7	2.8(0.7–4.9)
FQ+KNA	10	4.0(1.6–6.5)
FQ+AMK+CMP	1	0.4*
FQ+AMK+KNA	1	0.4*
FQ+CMP+KNA	1	0.4*
FQ+AMK+CMP+KNA	4	1.6*
FQ+AMK(2)/CPM(1)/KNA+PTA	3	1.2*
FQ+AMK+CMP/KNA+PTA	3	1.2*
FQ+AMK+CMP+KNA+PTA	1	0.4*

CI, confidence interval; INH, isoniazid; RFP, rifampin, SM, streptomycin, EMB, ethambutol; MDR, multidrug-resistant; FQ, fluoroquinolines (specifically ciprofloxacin, levofloxacin and ciprofloxacin.); AMK, amikacin; CPM, capreomycin; PTA: prothionamid, KAN: kanamycin.

* 95%CI was not determined.

### Resistance to the second-line drugs

Overall, isolates from 32.5% (95% CI, 30.4%–34.7%) patients were resistant to the second-line drugs. Fluoroquinolones had the highest resistance rate (25.2%; 95% CI, 23.3%–27.2%), followed by kanamycin (8.9%; 95% CI, 7.6%–10.2%), capreomycin (6.4%; 95% CI, 5.3%–7.5%), amikacin (6.0%; 95% CI, 5.0%–7.0%) and prothionamide (4.5%; 95% CI, 3.6%–5.4%). Isolates from 69 (3.6%, 95% CI, 2.8%–4.5%) isolates were resistant to ≥3 second-line drugs and 56 (2.96%; 95% CI, 2.2%–3.7%) fluoroquinolone monoresistance isolates were detected.

Among the 249 MDR TB patients, 77 (30.9%; 95% CI, 25.1%–36.7%) were identified as pre-XDR TB cases, 65 (26.0%), 6 (2.4%), 4 (1.6%) and 2 (0.8%) were resistant to fluoroquinolones, amikacin, kanamycin and capreomycin, respectively. Of the MDR TB patients, 31 (12.5%; 95% CI, 8.3%–16.6%) were XDR TB cases, among which, 10 patients were newly diagnosed with XDR rate of 11.2% and 13.1% for re-treatment patients; 17 patients were resistant to ≥6 routinely tested drugs (12 patients were other nationality and only 5 were Han peoples, and of 5 XDR TB patients who were resistant to 8 drugs: isoniazid, rifampin, ethambutol, streptomycin, fluoroquinolones, kanamycin, amikacin and capreomycin, 4 were other nationality group and 1 was resistant to all drugs test in the study), while the other patients were resistant to <6 drugs and 1 patient to all drugs test in this study (table 1).

### Characteristics of non-MDR, MDR/XDR, non-MXR MDR and XDR TB patients

The characteristics of patients with MDR/XDR (n = 249), non-XDR MDR (n = 218) and XDR TB (n = 31) are summarized and compared with those patients without MDR/XDR (n = 1644, table S1). No significant difference in the prevalence of MDR and XDR was observed among sex, age, ethnic group, patient type and treatment status. We found no significant differences between patients with XDR TB and patients with MDR but not XDR (non-XDR MDR) TB. There was a higher prevalence of XDR TB in other nationality (14.9%, 20/134) than in Chinese Han patients (9.6%, 11/115), although the difference did not attain significant level (*P* = 0.26, data not shown in the table S1).

To investigate the ethnic effects on heterogeneity of drug resistance, we stratified the patients into Han and non-Chinese Han sub-groups, and made further analysis on the ethnic specific estimation and comparison (table 2 and 3). Two strata were defined and the ethnic specific MDR/XDR prevalence was compared. After the stratification analysis, patients with MDR/XDR TB were significantly less likely to be younger (odds ratio [OR] 0.42 for age 20–29 years; 0.52 for age 40–49 years, table 2) with a *P*_trend_ value 0.004 (data not shown in the table 3), while absent for sex, patient type and treatment status in Chinese Han population. No significant differences between patients with XDR TB and patients with MDR but not XDR (non-XDR MDR) TB were observed (table 3).

**Table 2:** Comparison of characteristics between MDR and XDR prevalence in Chinese Han population.

Characteristics	No. (%) isolates				OR* (95%CI)		
	non-MDR	MDR/XDR	non-XDR MDR	XDR	MDR/XDR vs. non-MDR	XDR vs. non-XDR MDR	XDR vs. non-MDR
Sex							
Male	503(88.1)	68(11.9)	63(92.6)	5(7.4)	reference	reference	reference
Female	302(86.5)	47(13.5)	41(87.2)	6(12.8)	1.16(0.77–1.74)	1.97(0.54–7.23)	1.96(0.58–6.55)
Age group							
<20	53(86.9)	8(13.1)	8(100)	0(0.0)	0.66(0.29–1.50)	-	-
20–29	183(92.0)	16(8.0)	15(93.8)	1(6.3)	**0.40(0.21–0.73)**	0.37(0.04–3.56)	0.24(0.03–2.07)
30–39	128(88.9)	16(11.1)	15(93.8)	1(6.3)	0.57(0.30–1.05)	0.45(0.05–4.20)	0.30(0.03–2.53)
40–49	147(90.2)	16(9.8)	14(87.5)	2(12.5)	**0.50(0.27–0.92)**	0.95(0.17–5.42)	0.56(0.11–2.87)
50–59	83(84.7)	15(15.3)	14(93.3)	1(6.7)	0.86(0.45–1.64)	0.48(0.05–4.45)	0.48(0.06–4.09)
≥60	211(82.7)	44(17.3)	38(86.4)	6(13.6)	reference	reference	reference
Patients type							
Inpatients	668(87.2)	98(12.8)	90(91.8)	8(8.2)	reference	reference	reference
Outpatients	137(89.0)	17(11.0)	14(82.4)	3(17.6)	0.68(0.38–1.23)	1.72(0.38–7.78)	1.03(0.24–4.37)
TB treatment							
New	346(87.6)	49(12.4)	46(93.9)	3(6.1)	reference	reference	reference
Retreatment	459(87.4)	66(12.6)	58(87.9)	8(12.1)	1.03(0.68–1.56)	1.70(0.41–7.13)	1.82(0.45–7.40)

CI, confidence interval; MDR, multidrug resistance; MDR/XDR, MDR TB and XDR TB; non-XDR MDR, MDR without XDR; OR, odds ratio; XDR, extensively drug resistance; Boldface indicates significance.

* Adjusted for the characteristics for which adjusted ORs are shown.

**Table 3:** Comparison of characteristics between MDR and XDR prevalence in non-Chinese Han population.

Characteristics	No. (%) isolates				OR* (95%CI)		
	non-MDR	MDR/XDR	non-XDR MDR	XDR	MDR/XDR vs. non-MDR	XDR vs. non-XDR MDR	XDR vs. non-MDR
Sex							
Male	441(86.1)	71(13.9)	60(84.5)	11(15.5)	reference	reference	reference
Female	398(86.3)	63(13.7)	54(85.7)	9(14.3)	1.02(0.71–1.48)	0.89(0.32–2.48)	0.89(0.36–2.18)
Age group							
<20	70(94.6)	4(5.4)	4(100)	0(0)	0.46(0.15–1.39)	-	-
20–29	222(84.1)	42(15.9)	36(85.7)	6(14.3)	1.51(0.87–2.60)	0.71(0.18–2.85)	1.01(0.30–3.42)
30–39	158(84.5)	29(15.5)	25(86.2)	4(13.8)	1.44(0.80–2.61)	0.67(0.15–3.08)	0.96(0.25–3.72)
40–49	92(82.9)	19(17.1)	15(78.9)	4(21.1)	1.65(0.85–3.19)	1.00(0.21–4.82)	1.62(0.42–6.25)
50–59	106(86.2)	17(13.8)	16(94.1)	1(5.9)	1.43(0.73–2.82)	0.20(0.02–2.10)	0.32(0.04–2.79)
≥60	191(89.3)	23(10.7)	18(78.3)	5(21.7)	reference	reference	reference
Patients type							
Inpatients	728(85.4)	124(14.6)	107(86.3)	17(13.7)	reference	reference	reference
Outpatients	111(91.7)	10(8.3)	7(70.0)	3(30.0)	0.54(0.27–1.08)	3.85(0.77–19.23)	1.58(0.43–5.77)
TB treatment							
New	273(87.2)	40(12.8)	33(82.5)	7(17.5)	reference	reference	reference
Retreatment	566(85.8)	94(14.2)	81(86.2)	13(13.8)	1.20(0.80–1.79)	0.83(0.29–2.38)	0.87(0.34–2.22)

CI, confidence interval; MDR, multidrug resistance; MDR/XDR, MDR TB and XDR TB; non-XDR MDR, MDR without XDR; OR, odds ratio; XDR, extensively drug resistance; Boldface indicates significance.

* Adjusted for the characteristics for which adjusted ORs are shown.

## Discussion

Although XDR TB is a new term, cases have been occurring for over decade [9] and are now recognized worldwide [10]. It was reported that XDR TB prevalence among MDR TB cases ranged from 6.6% to 23.7% worldwide [11]. China has the second highest prevalence of TB and MDR TB burden, but the information on XDR TB is scant, which blocked treatment and control of TB to some extent. The most recent surveillance data from Beijing and Shanghai city and Shandong province in China showed that the XDR TB cases accounted for 6.3%–18.7% of MDR TB cases [12], [13], [14], [15], [16]. By analyzing first- and second-line drug resistance profiles of 1893 clinical *M. tuberculosis* isolates from Xinjiang Autonomous Region, we found that 13.2% TB patients met the definition for MDR, of which, 30.8% TB patients were pre-XDR, and 12.5% of MDR strains met the definition for XDR. However, the XDR TB is relatively higher than the previous surveillance data in Beijing and Shanghai but lower in Shandong province [12], [13], [14], [15], [16]. Our study also provided the age difference of the MDR/XDR TB distribution between Chinese Han and non-Chinese Han population, in which the MDR prevalence decreased with the age diminution compared with older population (*P*_trend_ = 0.004) inconsistent with previous report in Japanese population [17], while any association was not found in other nationality population.

According to recent Meta-analysis study for drug-resistant TB in China [18], the prevalence of MDR was estimated to be 5.3%, which is significant lower than the surveillance data in Beijing, Shanghai, Shandong and our study, also lower than the national surveillance data of MDR (8.3%). Our study indicated that the prevalence of MDR and XDR TB was 13.2% and 12.5% (among MDR TB), respectively. Of the XDR TB, the other nationality people (14.9%, 20/134) have a higher prevalence than Han people (9.6%, 11/115). Variety of different ethnic groups in genetic background, drug metabolism and life habits would be responsible for the difference.

We also identified an important subset of patients with pre-XDR TB, and majority of pre-XDR TB cases were resistant to fluoroquinolines (84.9%). In a study of South Africa using a convenience sample, 14% MDR isolates were found to be pre-XDR [19]. These findings suggest that we must implement strategies to identify and cure patients with pre-XDR TB before they develop XDR TB. Modeling studies support the notion that unless evolution of MDR TB into XDR TB is slowed, the number of XDR TB cases could increase exponentially [20].

Traditionally, TB control efforts were focused mainly on improvement of cure rates for drug-susceptible disease to reduce the number of drug-resistant cases arising from acquired resistance [21]. However, many health systems and national TB program have performed poorly during the past two decades due to recruit and train sufficient health workers [22] and to regulate drug suppliers and pharmacies [23]. Another important issue has been the failure to provide free treatment for drug-susceptible disease, which created ideal environment for acquisition of drug resistance [24]. Additionally, treatment costs for MDR TB was high, especially for these who lived in the village and low income patients, leading to similarly poor-quality treatment of MDR TB with second-line drugs, which has given rise to second-line drug resistance and emergence of XDR strains. China is a developing country, although the high speed increasing of economy in recent years. A relatively large regional difference in economic development is uneven, resulting in a high incidence of TB in rural areas than in the cities. Although the Chinese government in recent years implemented a new health care system, there is no enough money for them to go to a good hospital for medical treatment from the disease onset. Most TB patients from the rural areas were always received an intermittent treatment due to lack of money, increasing the possibility of the prevalence of drug-resistant strains. Evermore, the current standard care of TB patients in China (National Tuberculosis Program) does not include the first- and second-line anti-TB DST because of its prohibitive cost. All of these factors could confound the high prevalence of MDR and XDR TB in China.

The limitations of the current study should address. This study was carried out in only one specialized TB hospital in Xinjiang, the results might not reflect overall situation in the region; this hospital is the largest specialized hospital for TB in the region and provides medical care for TB patients of all ethnics throughout the region. There is no selection bias on the demographic characteristics of the patients. However, this hospital might have higher inclusion rate of serious TB cases than other hospitals in the region, whether this selection bias might lead to overestimation of drug resistant tuberculosis is unclear. Moreover, the outcome of XDR TB patients failed to be investigated due to the difficulty in following up of the patients.

In conclusion, the transmission of drug-resistant TB among Chinese population is extensive and widespread, the prevalence of MDR TB remains high, and the presence of pre-XDR and XDR TB will impose a new challenge in the control of TB. However, the ethnic difference would not affect the prevalence of MDR and XDR TB in Xinjiang Uygur Autonomous Region. Continuous surveillance of clinical isolates of *M. tuberculosis* is needed to identify MDR TB or XDR TB, especially in patients with a history of TB and those who have received prior anti-TB treatment.

## Materials and Methods

### Ethical Approval

Individual participants gave written informed consent before enrollment in the study. We obtained parental consent and patient assent for participants <18 years of age, and a parent served as a proxy for the interview of participants <13 years of age. The study was approved by the institutional review board at CHXUAR, China.

### Setting and study subjects

The study was conducted from June 2009 to June 2011 at the CHXUAR, which is the largest specialized hospital for TB in the region and provides medical care for >9% of the total TB patients throughout the region. CHXUAR is the provincial level reference laboratory and laboratory diagnosis center for TB, also the Revised National Tuberculosis Control Program accredited laboratory for DST. The laboratory participates and passes the annual external quality assessment (EQA) of regional level and national level, which made the laboratory accredited for the DST. All the patients with clinically diagnosed TB at both outpatient and inpatient settings were invited to participate the study. Three sputum samples were collected prior to treatment initiation for newly diagnosed patients and during the treatment for previously diagnose patients, which were subject to acid-fast bacilli smear microscopy detection and culture for *M. tuberculosis* Those who were confirmed to be culture positive were enrolled for further DST.

### Drug susceptibility testing

Sputum for this study was tested by optical microscopic analysis of Ziehl-Nielsen–stained smears and Middlebrook 7H11 agar and Mycobacterial Growth Indicator Tube (MGIT) 960 broth system (Becton Dickinson, Franklin Lakes, NJ, USA) culture. DST of positive cultures were performed by using MGIT 960 System at the following concentrations: isoniazid 0.2 µg/mL, rifampin 1.0 µg/mL, ethambutol 7.5 µg/mL, streptomycin 2.0 µg/mL, ofloxacin 2 µg/mL, levofloxacin 2 µg/mL, kanamycin 5.0 µg/mL, capreomycin 10 µg/mL, amikacin10 µg/mL, prothionamide 5 µg/mL, and ciprofloxacin 1.0 µg/mL. Because levofloxacin, ciprofloxacin and ciprofloxacin are fluoroquinolones with full cross-resistance, they were considered as the same family of anti-TB drugs and represented by fluoroquinolones in our analysis. MDR TB is defined as resistance to the two first-line drugs, rifampicin and isoniazid. XDR TB is resistant to rifampicin and isoniazid, plus any fluoroquinolone and one of the injectable second-line drugs (amikacin, capreomycin, or kanamycin) [3]. Pre-XDR TB was defined as TB with resistance to isoniazid and rifampin and either a fluoroquinolone or a second-line injectable agent but not both [25]. All the tests were performed at the CHXUAR TB reference laboratory.

### Data collection and statistical analysis

Medical records of the enrolled patients were reviewed for demographic and clinical data. Information was obtained on sex, age, ethnic, previous history of TB, present address and medical records (such as presence of HIV infection, chest radiograph findings). The continuous variables were expressed as group means ± standard deviation (SD). The proportion was described by using a frequency with its 95% confidence interval (CI) by single-sample *t* test. Chi square test or Fisher's exact test was used for inter group comparison as appropriate. In bivariate analysis were included in a multivariate logistic regression model. Two-tailed *P* value of less than 0.05 was considered to be statistically significant.

## Supporting Information

Table S1 **Demographic and clinical characteristics between XDR TB and non-XDR MDR TB from Xinjiang, June 2009–June 2011.**
(DOC)
