# Supplementary material for: Multidrug-Resistant and Extensively Drug-Resistant Tuberculosis in Multi-Ethnic Region, Xinjiang Uygur Autonomous Region, China
Source: PLoS One. 2012 Feb 22;7(2):e32103. doi: 10.1371/journal.pone.0032103 (PMC3285197; doi:10.1371/journal.pone.0032103)
Supplement: Table S1 — Demographic and clinical characteristics between XDR TB and non-XDR MDR TB from Xinjiang, June 2009–June 2011. (DOC) [file pone.0032103.s001.doc]

Supplementary table 1 Demographic and clinical characteristics between XDR TB and non-XDR MDR TB from Xinjiang, June 2009-June 2011.

| Characteristics | No. (%) isolates | | | | | OR^*^ (95%CI) | | | |
| --- | --- | --- | --- | --- | --- | --- | --- | --- | --- |
|  | Non-MDR  n = 1644 | MDR/XDR  n = 249 | non-XDR MDR  n = 218 | XDR  n = 31 |  | | MDR/XDR vs.  Non-MDR | XDR vs. non-XDR  MDR | XDR vs.  Non-MDR |
| Sex |  |  |  |  |  | |  |  |  |
| Male | 944(87.2) | 139(12.8) | 123(88.5) | 16(11.5) |  | | reference | reference | reference |
| Female | 700(86.4) | 110(13.6) | 95(86.4) | 15(13.6) |  | | 1.08(0.82-1.42) | 1.30(0.58-2.92) | 1.15(0.56-2.36) |
| Age group |  |  |  |  |  | |  |  |  |
| <20 | 123(91.1) | 12(8.9) | 12(100) | 0(0.0) |  | | 0.55(0.28-1.04) | - | - |
| 20-29 | 405(87.5) | 58(12.5) | 51(87.9) | 7(12.1) |  | | 0.79(0.54-1.16) | 0.58(0.19-1.80) | 0.63(0.24-1.69) |
| 30-39 | 286(86.4) | 45(13.6) | 40(88.9) | 5(11.1) |  | | 0.86(0.57-1.30) | 0.49(0.14-1.69) | 0.64(0.22-1.90) |
| 40-49 | 239(87.2) | 35(12.8) | 29(82.9) | 6(17.1) |  | | 0.83(0.54-1.30) | 0.92(0.29-2.93) | 1.02(0.37-2.83) |
| 50-59 | 189(85.5) | 32(14.5) | 30(93.8) | 2(6.3) |  | | 1.03(0.65-1.63) | 0.26(0.05-1.34) | 0.35(0.08-1.62) |
| ≥60 | 402(85.7) | 67(14.3) | 56(83.6) | 11(16.4) |  | | reference | reference | reference |
| Ethnic group |  |  |  |  |  | |  |  |  |
| Han | 805(87.5) | 115(12.5) | 104(90.4) | 11(9.6) |  | | reference | reference | reference |
| Uygur | 644(87.0) | 96(13.0) | 82(85.4) | 14(14.6) |  | | 1.04(0.78-1.40) | 1.93(0.78-4.80) | 1.62(0.72-3.62) |
| Kazakh | 98(81.7) | 22(18.3) | 20(90.9) | 2(9.1) |  | | 1.57(0.95-2.60) | 1.22(0.23-6.39) | 1.55(0.33-7.22) |
| Hui | 76(86.4) | 12(13.6) | 9(75.0) | 3(25.0) |  | | 1.11(0.58-2.10) | 4.08(0.85-19.50) | 3.05(0.83-11.26) |
| Mongolian | 21(84.0) | 4(16.0) | 3(75.0) | 1(25.0) |  | | 1.33(0.45-3.95) | 16.79(0.86-327.06) | 4.23(0.51-35.02) |
| Patients type |  |  |  |  |  | |  |  |  |
| Inpatients | 1396(86.3) | 222(13.7) | 197(88.7) | 25(11.3) |  | | reference | reference | reference |
| Outpatients | 248(90.2) | 27(9.8) | 21(77.8) | 6(22.2) |  | | 0.62(0.40-0.97) | 2.63(0.88-7.89) | 1.40(0.54-3.64) |
| TB treatment |  |  |  |  |  | |  |  |  |
| New | 619(87.4) | 89(12.6) | 79(88.8) | 10(11.2) |  | | reference | reference | reference |
| Retreatment | 1025(86.5) | 160(13.5) | 139(86.9) | 21(13.1) |  | | 1.12(0.84-1.49) | 1.06(0.45-2.53) | 1.11(0.51-2.41) |

CI, confidence interval; MDR, multidrug resistance; MDR/XDR, MDR TB and XDR TB; non-XDR MDR, MDR without XDR; OR, odds ratio; XDR, extensively drug resistance; Black bold fonts represents P value less than 0.05. Boldface indicates significance.

* Adjusted for the characteristics for which adjusted ORs are shown.
